# Supplementary material for: Detection of Breast Cancer Cells Using Acoustics Aptasensor Specific to HER2 Receptors
Source: Biosensors (Basel). 2019 May 27;9(2):72. doi: 10.3390/bios9020072 (PMC6627288; doi:10.3390/bios9020072)
Supplement: Supplementary file 1 [file biosensors-09-00072-s001.pdf]

Supplementary Material

Article

Detection of Breast Cancer Cells Using Acoustics Aptasensor Specific to HER2 Receptors

Alexandra Poturnayová [1,2], Ľudmila Dzubinová [2], Monika Buríková [3], Jozef Bízik [3] and Tibor Hianik [2,*]

[1] Institute of Animal Biochemistry and Genetics, Center of Biosciences SAS, Dúbravská cesta 9, 840 05 Bratislava, Slovakia; alexandra.poturnayova@savba.sk
[2] Department of Nuclear Physics and Biophysics, Faculty of Mathematics, Physics and Informatics, Comenius University, Mlynská dolina F1, 842 48 Bratislava, Slovakia; ludmila.valigurska@gmail.com
[3] Cancer Research Institute, Biomedical Research Center SAS, Dúbravská cesta 9, 840 05 Bratislava, Slovakia; monika.burikova@savba.sk (M.B.); jozef.bizik@savba.sk (J.B.)
* Correspondence: tibor.hianik@fmph.uniba.sk; Tel.: +421-260-295-683

Received: 23 April 2019; Accepted: 23 May 2019; Published: 27 May 2019

S1. The kinetics of the changes of resonant frequency and motional resistance following addition of the cells on the surface of TSM transducer of various modifications

In this part we included kinetics of the changes of resonant frequency, Δf_s, and motional resistance, ΔR_m, following additions of HER2+ (SK-BR-3) and HER2- (MDA-MB-231) (B) cells at the surface of the biosensor based on HeA2_3 aptamers (Figure S1) as well as those formed by non-specific sgc8c aptamers (Figure S2). It can be seen that significant changes of the measuring values were observed only in the case of the interaction of SK-BR-3 cells with the surface of the sensor composed of specific DNA aptamers HeA2_3 (Figure S1A).

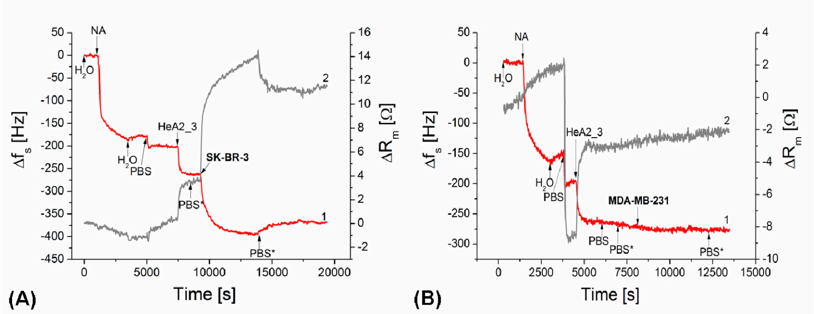

Figure S1. The kinetics of the changes of the series resonant frequency, Δf_s, (**curve 1**) and motional resistance, ΔR_m, (**curve 2**) of TSM transducer following addition of neutravidin (NA) (125 μg/mL), HeA2_3 aptamers (0.5 μM) and SK-BR-3 cells (**A**) or MDA-MB-231 cells (**B**). The concentration of the cells was 5 × 10^5 cells/mL. The arrows indicate addition of respective compounds as well as the washing of the surface by water, PBS or by PBS with addition of aliquot volume of DMEM (PBS*).

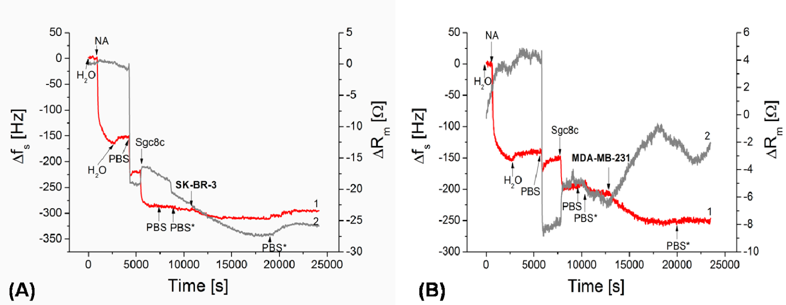

Figure S2. The kinetics of the changes of the series resonant frequency, Δf_s, (**curve 1**) and motional resistance, ΔR_m, (**curve 2**) of TSM transducer following addition of neutravidin (NA) (125 μg/mL), sgc8c aptamers (0.5 μM) and SK-BR-3 cells (**A**) or MDA-MB-231 cells (**B**). The concentration of the cells was 5 × 10^5 cells/mL. The arrows indicate addition of various compounds and the washing of the surface by water, PBS and by PBS with addition of aliquot volume of DMEM (PBS*).

Figure S3 demonstrates example of non specific interaction of SK-BR-3 cells with the surface of piezocrystal covered only by neutravidin. It can be seen that addition of the cells did not caused significant changes of resonant frequency. Only slight increase of motional resistance took place.

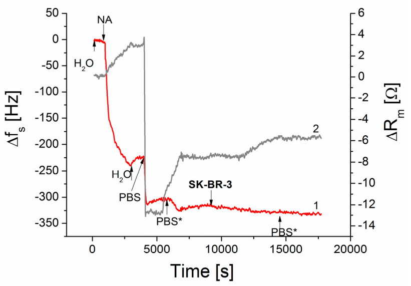

Figure S3. The kinetics of the changes of the series resonant frequency, Δf_s, (**curve 1**) and motional resistance, ΔR_m, (**curve 2**) of TSM transducer following addition of neutravidin (NA) (125 µg/mL) and SK-BR-3 cells in a concentration of 5 × 10^5 cells/mL. The arrows indicate addition of various compounds and the washing of the surface by water, PBS and by PBS with addition of aliquot volume of DMEM (PBS*).

S2. The topography of the of neutravidin layers studied by atomic force microscopy

In order to provide more detailed insight on the properties of the neutravidin layers at a gold support, we studied their topography using atomic force microscopy (AFM). The experiments were performed by means of an Agilent 5500 AFM (Agilent Technologies, USA) and silicon cantilevers (model MSCT, Bruker, Germany) with a nominal spring constant of 0.01 to 0.03 N/m. The detailed description of the measuring procedure can be found in our recent paper [1]. Figure S2-1 shows the topography of the thin gold layers of TSM transducer and those with immobilized neutravidin. The immobilization of neutravidin has been performed at the same conditions like those for preparation of sensing surfaces in TSM experiments described in the Section 2.4 (see the main text) including all washing steps. The figure S4 shows also the height profile of the surfaces.

The topography of freshly cleaned gold surface (Figure S4a) and their height profile (Figure S4c) revealed rather smooth layer with the roughness of R_{rms} = 1.66 nm. Miller at al. reported for analogically prepared gold layer the values of R_{rms} in a range of 0.2–1 nm [2]. AFM scan, however, shows that the gold layer is at certain parts interrupted by defects, which can be due to the cleaning by aggressive Piranha solution. This phenomenon is known from the literature [3]. The roughness of the neutravidin layers (Figure S4d) was R_{rms} = 2.09 nm, which is comparable with the results of Vermette et al. [4] that reported the value R_{rms} = 1.99 nm. Neutravidin forms rather smooth layer at the gold surface. Therefore, comparison of the R_{ms} values for gold and neutravidin layers demonstrates not substantial differences. In order to approve that neutravidin forms stable layer we applied mechanical scratching. The corresponding results are presented on Figure S5. This experiment is a clear evidence of the stability of neutravidin layer at gold surface. The thickness of this layer is according to the height profile (Figure S5, right panel) approx. 5 nm. This agree well with the neutravidin dimensions. Neutravidin is tetramer of quadratic shape with dimensions of 5.6 × 5.0 × 4.0 nm [5,6]. Thus, the obtained images provide evidence of the stability of the neutravidin layers, which supports well the results of TSM experiments that demonstrated stability of these layers after washing with deionised water and PBS. The stability of neutravidin layers at the gold surface has been approved also by our previous study of the neutravidin layers based aptasensors by surface plasmon resonance (SPR) method [7].

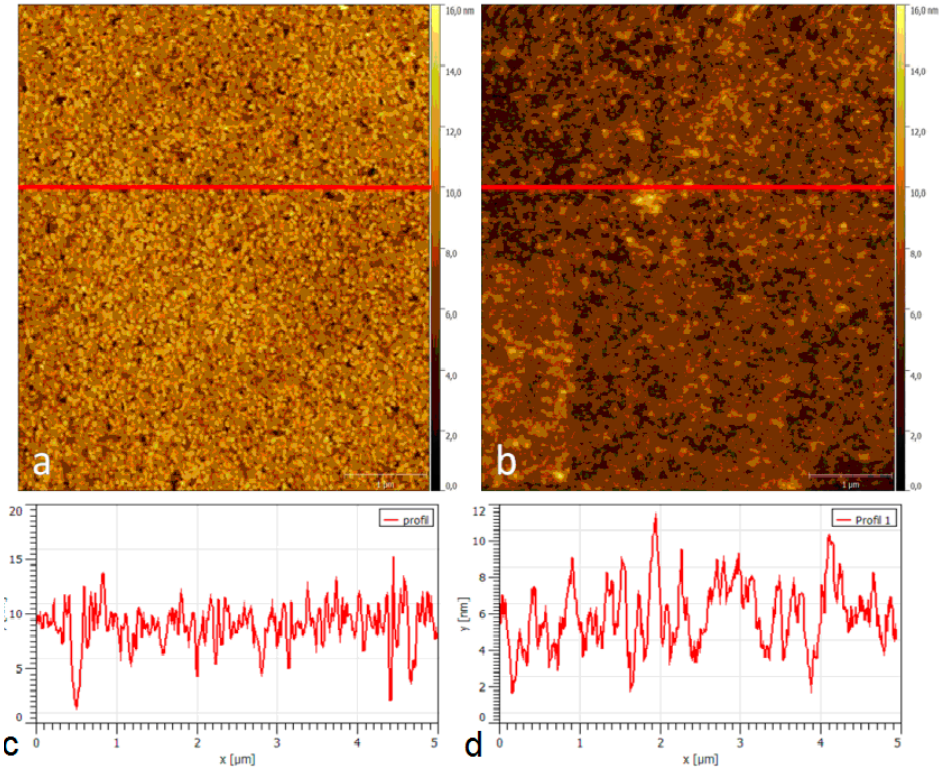

Figure S4. AFM topography of the surface of TSM transducer and the height profiles at marked part of the images for freshly cleaned gold layer (a,c) and for neutravidin immobilized at the gold layer (b,d). The scan size is 5 × 5 µm.

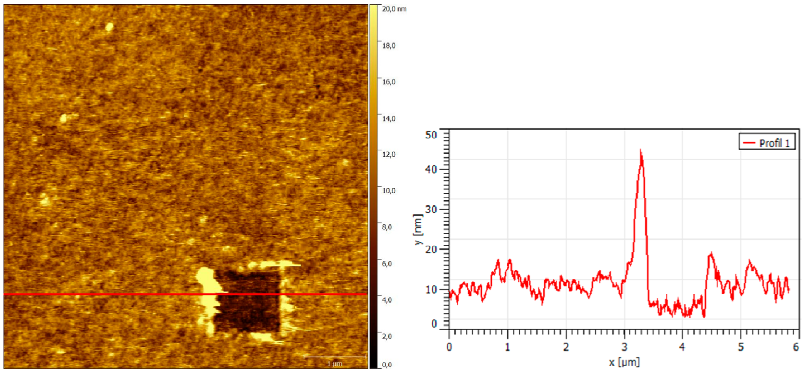

Figure S5. AFM topography of the neutravidin layer at gold after application of mechanical scratching (**left**) and the height profile at the marked site (**right**). The scan size is 6 × 6 µm.

References

1. Poturnayova, A.; Burikova, M.; Bizik, J.; Hianik, T. DNA Aptamers in the Detection of Leukemia Cells by the Thickness Shear Mode Acoustics Method. *ChemPhysChem* **2019**, *20*, 545 –554,.
2. Ferrato, M.-A.; Niec, A.; Carmichael, T.B.; Miller, M.S.; Biesinger, M.C. Ultrasmooth Gold Surfaces Prepared by Chemical Mechanical Polishing for Applications in Nanoscience. *Langmuir* **2014**, *30*, 14171–14178.
3. Bailey, T.; Choi, B.J.; Colburn, M.; Meissl, M.; Shaya, S.; Sreenivasan, S.V.; Willson, C.G.; Ekerdt, J.G. Step and flash imprint lithography: Template surface treatment and defect analysis. *J. Vac. Sci. Technol. B: Microelectron. Nanometer Struct.* **2000**, *18*, 3572.

4. Vermette, P.; Gengenbach, T.; Divisekera, U.; A Kambouris, P.; Griesser, H.J.; Meagher, L. Immobilization and surface characterization of NeutrAvidin biotin-binding protein on different hydrogel interlayers. *J. N.a. N.a. Sci.* **2003**, *259*, 13–26.
5. Rosano, C.; Arosio, P.; Bolognesi, M. The X-ray three-dimensional structure of avidin. *Biomol. Eng.* **1999**, *16*, 5–12.
6. Tsortos, A.; Papadakis, G.; Mitsakakis, K.; Melzak, K.A.; Gizeli, E. Quantitative Determination of Size and Shape of Surface-Bound DNA Using an Acoustic Wave Sensor. *Biophys. J.* **2008**, *94*, 2706–2715.
7. Ostatná, V.; Vaisocherová, H.; Homola, J.; Hianik, T. Effect of the immobilisation of DNA aptamers on the detection of thrombin by means of surface plasmon resonance. *Anal. Bioanal. Chem.* **2008**, *391*, 1861–1869.

© 2019 by the authors. Licensee MDPI, Basel, Switzerland. This article is an open access article distributed under the terms and conditions of the Creative Commons Attribution (CC BY) license (http://creativecommons.org/licenses/by/4.0/).
